# Light responsive hydrogels for controlled drug delivery

**DOI:** 10.3389/fbioe.2022.1075670

**Published:** 2022-12-16

**Authors:** Yanghui Xing, Buhui Zeng, Wang Yang

**Affiliations:** Department of Biomedical Engineering, Shantou University, Shantou, China

**Keywords:** hydrogel, light responsive, drug delivery, photosensitizer, programmed release

## Abstract

Light is an easy acquired, effective and non-invasive external stimulus with great flexibility and focusability. Thus, light responsive hydrogels are of particular interests to researchers in developing accurate and controlled drug delivery systems. Light responsive hydrogels are obtained by incorporating photosensitive moieties into their polymeric structures. Drug release can be realized through three major mechanisms: photoisomerization, photochemical reaction and photothermal reaction. Recent advances in material science have resulted in great development of photosensitizers, such as rare metal nanostructures and black phosphorus nanoparticles, in order to respond to a variety of light sources. Hydrogels incorporated with photosensitizers are crucial for clinical applications, and the use of ultraviolet and near-infrared light as well as up-conversion nanoparticles has greatly increased the therapeutic effects. Existing light responsive drug delivery systems have been utilized in delivering drugs, proteins and genes for chemotherapy, immunotherapy, photodynamic therapy, gene therapy, wound healing and other applications. Principles associated with site-specific targeting, metabolism, and toxicity are used to optimize efficacy and safety, and to improve patient compliance and convenience. In view of the importance of this field, we review current development, challenges and future perspectives of light responsive hydrogels for controlled drug delivery.

## 1 Introduction

Hydrogels are three-dimensional crosslinked polymeric networks that can absorb large amounts of water or biological fluids. Their structures are formed through chemical or physical crosslinking of different polymer chains, between which either covalent bonds or physical interactions exist to maintain their structural stability (Ebara et al., 2014). Hydrogels are usually characterized with pH-neutral, colorless, odorless, non-toxic, high absorption ability, and excellent stability and constancy in storage with controllable biodegradability (Ullah et al., 2015). Given their technical features, hydrogels have been utilized in designing drug delivery systems for years. Hydrogels can protect the drug from surrounding environments, and their tunable properties plus the ability to retain large fraction of solvents make them ideal carriers for drug delivery systems. By changing hydrogel properties, drug release rate can be accurately controlled. Additionally, hydrogels usually have low affinity with drugs, thus achieve a high fraction of drug release.

Hydrogels can be classified into two groups according to their responses to external stimuli: one is conventional hydrogels, which have no particular sensation to changes of their environment, and the other is stimuli-responsive hydrogels, also known as smart hydrogels that are capable of responding to physical, chemical or biochemical stimuli (Ji et al.eng, 2020). In response to external stimuli, these hydrogels undergo a series of changes in their growth actions, network structure, mechanical strength and permeability (Zhang et al., 2021; Gil and Hudson, 2004/12). Stimuli-responsive hydrogels contain specific components as “on-off” sensors able to detect stimulation signals and subsequently control their changes. For example, ultrasound amplitude and time duration are associated FITC-BSA release rate in chitosan hydrogels with reversible Diels-Alder linkers (Arrizabalaga et al.eng, 2022); and thermosensitive mPEG-PA-PLL hydrogel was used for controlled oral delivery of calcitonin (Cheng et al., 2022). Combining two or more stimuli responsive mechanisms in one hydrogel system, multi-responsive hydrogels can be formed to respond to more than two external stimuli. PF127/TMC/PEG-HA can react to both pH and temperature, and is an example of a dual responsive hydrogel used for in textile-based transdermal therapy (Chatterjee et al., 2019).

## 2 Fundamentals of light responsive drug delivery system

Light responsive hydrogels are able to respond to light irradiation and subsequently give rise to structural and conformational changes (Jiang and Wen, 2015; Bustamante-Torres et al., 2021). Light is an easy acquired, effective and non-invasive external stimulus that can be highly focused and regulated by manipulating its parameters, including intensity, wavelength, exposure time and beam diameter. Consequently, light offers accurate and spatiotemporal control of drug delivery, and thus received great attention in the past decade (Li et al., 2018a; Rizzo et al., 2021). Light responsive hydrogels are obtained by incorporating photosensitive moieties into the polymer structure. Based on the photosensitizer (PS) used, the response can be reversible or irreversible (Bustamante-Torres et al., 2021). Light can cause cleavage, isomerization, or dimerization of photosensitive groups in hydrogels, and leads to partial or complete decrosslinking, degradation, swelling and/or shrinkage of the hydrogel structure.

**FIGURE 1:**
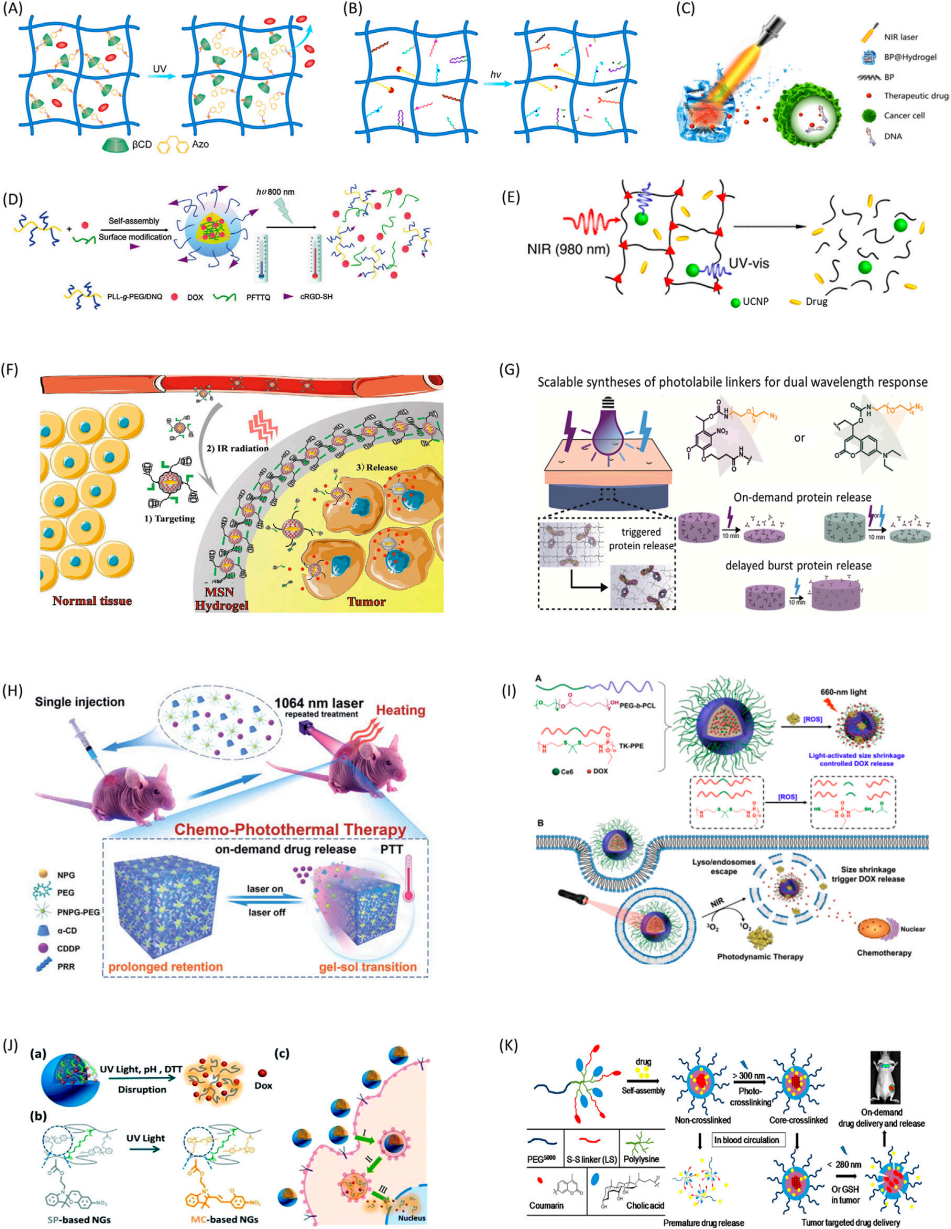
Representative mechanisms and applications of light-controlled drug delivery systems. **(A)** Photoisomerization induced drug release through guest-host interactions. **(B)** Photocleavage induced drug release through multi mechanisms. **(C)** Photothermal effect-induced drug release to break DNA chains. Copyright Qiu et al. (2018) NAS. **(D)** Two-photon light regulated chemo-photothermal therapy with fast drug release. Used with permission of RSC from Yuan et al. (2015). **(E)** NIR-triggered hydrogel degradation using the UV light generated by UCNPs. Adapted with permission from Yan et al. Copyright 2012 American Chemical Society. **(F)** Light-induced targeted drug delivery to tumor tissue through photoisomerization. Adapted with permission from Chen et al. Copyright 2016 American Chemical Society. **(G)** On-demand protein release by dual wavelength lights. Adapted with permission from LeValley et al. Copyright 2020 American Chemical Society. **(H)** NIR induced repetitive on-demand drug release for chemo-photothermal therapy. Ruan et al. (2019). **(I)** Combination of photodynamic therapy and chemotherapy through light-induced drug release. Adapted with permission from Cao et al. Copyright 2018 American Chemical Society. **(J)** DOX release process from light-induced multi stimuli responsive system. Used with permission of RSC from Chen et al., 2017. **(K)** Light and redox dual responsive coumarin containing micelles as drug nanocarrier for cancer therapy. Shao et al. (2014).

The light-controlled drug delivery systems can be classified into three broad categories: photoisomerization-based, photochemical-based, and photothermal-based drug release platforms. Photoisomerization typically involves conformational changes of the hydrogels from *trans* to *cis* under light irradiation. During this process, hydrogels open pore sizes and allow drugs to diffuse out of their matrixes (Figure 1A). It does not break chemical bonds of hydrogels, and the process is usually reversible and repeatable (Tomatsu et al., 2011). Photochemical reactions can lead to network structure and configuration changes of the hydrogel, and subsequently induce drug release (Iwaso et al., 2016). Among all photochemical reactions, photocleavage is the one of most commonly used ones for controlled drug delivery. This method is realized by incorporating photocleavable linkers into the hydrogel structure to create nanoparticles that can be cut off by light (Figure 1B) (Miranda and Lovell, 2016; Liese and Hampp, 2011/04). In this case, drugs are tethered to the hydrogel network at designed or selected sites covalently through photocleavable linkers, and can maintain their efficacy and avoid unwanted release to a large extent (Shadish and DeForest, 2020; Wang et al., 2020). The photothermal reaction is utilizing materials able to convert light energy into the heat energy, which in turn induces a disruption of a thermally sensitive drug carrier (Figure 1C) (Qiu et al., 2018; Bordbar-Khiabani and Gasik, 2022). The reaction requires two components - a photosensitizer to convert light into heat, and a material sensitive to temperature changes for drug release.

The commonly used light sources include ultraviolet (UV), visible light and near-infrared (NIR). UV once was the most popular light sources because of the availability of a wide range of UV photosensitizers. But it is mainly for *in vitro* experiments due to its cytotoxicity and low tissue penetration. On the side, NIR is safer for *in vivo* studies and able to trigger drug release within deep tissues. Thus, NIR is very attractable for clinical applications. Due to recent advance of material science, many new photosensitizers responsive to NIR were developed. Additionally, the use of two-photon excitation (Figure 1D) and up-conversion nanoparticles (UCNPs) (Figure 1E) also greatly expanded situations that NIR can be used (Pokharel and Park, 2022; Yuan et al., 2015; Yan et al., 2012/10). Based on above mechanisms, a variety of drug delivery applications were presented in Table 1 (Tao et al., 2019; Liang et al.eng, 2020; Pourjavadi et al., 2020; Wei et al., 2020; Yu et al., 2020).

**TABLE 1:** Representative light-controlled drug delivery systems.

Delivery systems	Stimuli and drug release mechanisms	Applications	Highlights	Reference
PEG-Azo2 with alginate-βCD	UV/photoisomerization with guest-host chemistry	Release of small molecules for tissue engineering and wound healing	Light induced rapid and controlled release of small molecules, suitable for wound healing	Chiang and Chu, (2015)
HA with Azo and βCD	UV/photoisomerization with guest-host chemistry	BSA release in cell culture environments	Reversible guest-host interactions and accurate control of dug release	Rosales et al. (2018)
PAA with mAzo and βCD	Red light/photoisomerization with guest-host chemistry	*in vivo* protein release	Controlled deep tissue drug delivery	Wang et al. (2015)
SPMA with spiropyran–merocyanine	UV/photoisomerization induced volumetric change	*in vitro* DOX release	On-demand reversible drug release with hydrophobicity switch	(Ghani et al., 2021/01)
PAA, spiropyran, disulfide-containing cystamine	UV/photoisomerization resulted multi stimuli response	*in vitro* DOX release	Light, pH, and redox triple-responsive nanogel	Chen et al. (2017)
PEGMA with tethered exosomes	Blue light/photocleavage	BMP loaded exosome release in cells to deliver small molecules	Controlled release through hydrogel structure, efficient cell uptake	Yerneni et al.eng, (2022)
Injectable glycol chitosan with IR783-mHNK	NIR/photocleavage	*in vivo* mHNK release in mice	Accurate control, good biocompatibility and stability, minimum leakage and efficient light responsibility	Yang et al. (2022)
PEG-SH, S,S-Tetrazine	Green light/photocleavage - light induced gel degradation	*in vivo* DOX release in mice for cancer therapy	Drug release with hyperoxide-accelerated behaviors and antitumor effects	Wang et al. (2020)
PEG, polylysine, coumarin	UV/photocleavage induced light and redox responses	*in vivo* drug release for cancer therapy	Good drug loading capacity and stability, preferred tumor accumulation and the prolonged tumor residency	Shao et al. (2014)
PVA pBP composite	NIR/photothermal effects	*in vitro* congo red release	Robust mechanical properties, excellent biocompatibility, highly controllable drug release	Yang et al. (2018)
Oxidized dextran and platinum nanoparticles	NIR/photothermal effects	*in vitro* and *in vivo* drug release for cancer therapy	Long-term repeated PTT with excellent photothermal effects and good biocompatibility	Li et al. (2018b)
Agarose, HK ink, dihydroartemisinin	NIR-II/photothermal effects and reversible gel degradation	*in vivo* release of drug targeting tissues with pre-injected DHA	Injectable, deep tissue penetration, accurate tissue targeting	Chen et al. (2020)
GelPV-DOX-DBNP	NIR and red light/hydrogel degradation and photothermal effects	*in vivo* DOX release for cancer therapy	Combined chemo-photothermal therapy with two-step accurate control of drug release	Sun et al.eng, (2020)
Chitosan/PLA/PNIPAM Hydrogels coated Gold Nano Rods	NIR/hydrogel volumetric shrinkage due to photothermal effects	*In vitro* study of paclitaxel (PTX) delivery to cells	Multi-stimuli sensitive systems able to respond to light, heat, and pH	Pourjavadi et al. (2020)
PLGA nanoparticles with Graphene Quantum Dots or Methylene Blue	NIR/hydrogel degradation due to photothermal effects	Chemo-photothermal or chemo-photodynamic therapy for cancer	Combined chemotherapy with PTT and PDT, as well as accurate release control	Liang et al.eng, (2020)
NIPAm, MPCD with gold nanorods (GNRs)	NIR/photothermal and pH responsive effects	Chemophotothermal synergistic cancer therapy	Good mechanical and swelling properties, gelation characteristics, and excellent NIR-responsive property	Xu et al.eng, (2017)
PNPG-PEG-aCD	NIR-II/photothermal effects	Cisplatin release for *in vivo* chemo-photothermal therapy	Repeatable and accurate controlled drug release, deep tissue penetration	Ruan et al. (2019)
Gelatin, PDA and alginate	NIR/photothermal effects	Localized therapy of breast cancer	3D printed scaffolds for accurate structure control and drug release	Wei et al. (2020)
PLGA coated Au-TiO2	NIR/photothermal effects	Human papillary thyroid carcinoma therapy	High efficiency, good biocompatibility, accurate control	Yu et al. (2020)
Gel-MA, BACA with Cu NPs	NIR/photothermal effects and ROS production	Skin tissue regeneration	Multifunctional hydrogel for killing bacteria and accelerating wound healing	Tao et al. (2019)

## 3 Photoisomerization-based drug delivery systems

Photoisomerization takes advantage of the conformational changes of certain molecules when stimulated by light irradiation (Pan et al., 2021/10). Azobenzene is the most used molecules in this category. Modifying hydrogels with azobenzene and cyclodextrin (CD) can create light responsive crosslinking between the two components. When in the *trans* isomer form, azobenzene can have “host-guest” interaction with CD, forming strong crosslinks. Under UV light irradiation, the azobenzene changes to *cis* isomer form which leads to breaking of crosslinks and drug release.

Light responsive azobenzene-CD guest-host chemistry has been widely used for controlled release of drugs from hydrogels under light exposure (Rosales et al., 2018). In one study, the authors developed a light responsive hydrogel by permeating diazobenzene-modified poly(ethylene glycol) (PEG) into βCD grafted alginate. Exposure of UV light leads to controlled cargo release from the hydrogel which was used in wound healing and other applications (Chiang and Chu, 2015). In another study, a model drug was released from a light responsive hydrogel made from azobenzene and PEG. The system showed reversible photoisomerization between its *cis* and *trans* isomers under UV-light irradiation allowing model drug release from the hydrogel network. The authors observed clear volume changes during the photoisomerization process and characterized the peak effects at 330 nm and 435 nm respectively. Thus, the drug release can be controlled by the wavelength and intensity of light irradiation (Rastogi et al.eng, 2018). Additionally, Nehls et al. showed similar results with more accurately controlled release rate of an entrapped model drug based on azobenzene-CD chemistry in PEG hydrogels (Nehls et al., 2016). Furthermore, PAA-based hydrogel modified with methoxy-substituted azobenzene and βCD supramolecular complexes showed a gel to sol transition in response to red light irradiation, and subsequently resulted in a dose dependent manner of loaded BSA release. With higher wavelength than UV, red light can be used for deep tissue drug delivery with less energy-induced damage (Wang et al., 2015). Chen et al. designed a DOX-loaded delivery system targeted tumor tissues using muli-responsive formation and degradation of the hydrogel (Figure 1F) (Chen et al.eng, 2016).

Spiropyran is another well-known photosensitizer that can be incorporated into hydrogel networks. It was used to deliver a variety of drugs such as doxorubicin and paclitaxel based on photoisomerization of hydrophobic spiropyran to hydrophilic merocyanine after UV irradiation. During this process, the swelling of hydrogels cause water soluble drugs to diffuse out of the hydrogel networks (Li et al., 2020/05). Tong et al. reported a nanoparticulate drug delivery system comprising spiropyran and PEGylated lipid that allows repetitive drug delivery at given time and location. The light-sensitive switch enables particles to fluoresce and release drugs inside cells when illuminated with UV light providing a spatiotemporal control of drug delivery with and enhanced tissue penetration (Tong et al., 2012). Spiropyrans within hydrogels have also been used as on and off switch triggers for controlled drug release in a number of other studies due to its reversible properties (Ghani et al., 2021/01; Xiao et al.eng, 2016).

## 4 Photochemical reaction-based drug delivery systems

Photochemical reactions include photooxidation, photocleavage and photopolymerization. For photocleavage induced drug release, o-nitrobenzyl is the most popular photocleavable linkers. It can be incorporated into many hydrogels to give them light responsive properties. The cleavage of o-nitrobenzyls happens at C-O bond in its ester group after exposure to UV light or high energy visual light. In one study, o-nitrobenzyl moieties were added into gelatin methacryloyl(-acetyl) hydrogels with a biotin-functionalized photocleavable macromer, and then controlled release under UV-irradiation is studied. The authors found that liquid chromatography coupled to mass spectrometry analysis of aqueous linker solutions allows the identification of the main cleavage products and the cleavage kinetics (Claaßen et al., 2018). O-nitrobenzyls were also used to cause a macro physical change in the overall structure for drug delivery (Linsley et al., 2017). In o-nitrobenzyl linked PEG and PAM hydrogels, cleavage of o-nitrobenzyls with UV resulted volume shrinking of their polymeric structure, and caused controlled release of drugs entrapped within its matrix (Yan et al., 2012/10). In a similar study for PEG and dextran hydrogel with o-nitrobenzyl linkers, 60 min exposure to UV leads to fifty percent of model drug release due to hydrogel structure dissociation (Peng et al., 2011). In another study, authors showed a novel strategy enabling the use of upconversion luminescence converting NIR light into UV light, which are received by o-nitrobenzyl groups in PEG hydrogels. Subsequently, the photocleavage reaction leads to tethered drugs (Yan et al., 2011/12).

A ruthenium-based photocleavable linker was developed to form hydrogel with entrapped model drugs, which cannot be released until exposure to light. By varying the coordinated ligands, Ru-cross-linkers have 1-photon absorption maxima that are tunable across the visible spectrum and into the near-infrared, which enables photoactivation at multiple, different wavelengths (Rapp and Dmochowski, 2019). In a more recent study, Yerneni et al. tethered exosomes to poly(ethylene oxide)-based hydrogels using atom transfer radical polymerization. The method allowed controlled release over a period of 1 month and the release profile can be programmed through crosslinking density and light stimuli conditions (Yerneni et al.eng, 2022). Yang et al. developed a photocleavable prodrug loaded injectable glycol chitosan (GC) hydrogel for NIR-triggered repetitive drug release. The hydrogel shows good stability, minimum leakage and efficient light responsibility both *in vitro* and *in vivo* (Yang et al., 2022).

When photocleavable linkers attach model drugs to polymer matrix covalently, it may cause great reduction to drug activities as well as unspecified tethers. To overcome these disadvantages, some researchers used recombinant protein techniques to modify proteins with a handle in order to attach a protein of interest to the hydrogel matrix. In one study, a PEG-based hydrogel was developed *via* a click reaction without impact on entrapped enzyme activity under 60°C thermal stress for weeks. The study showed a general method to preserve drug activities at certain conditions and enable controlled drug release when needed (Sridhar et al., 2018/03). In another study, the authors developed a technique to use light-sensing proteins as light-activated reversible binding sites within synthetic poly(ethylene glycol) (PEG) hydrogels. It has reversible changes between “light” and “dark” conformations in response to different lights to control a recombinant protein release from PEG hydrogels spatiotemporally (Hammer et al., 2020/07). In a recent study, both nitrobenzyl and coumarin were used for photolabile crosslinks, and subsequently, on-demand and tunable dual wavelength release of antibody was achieved (Figure 1G) (LeValley et al.eng, 2020).

## 5 Photothermal reaction-based drug delivery systems

Photothermal therapy-based drug delivery system includes a photothermal agent, which is able to generate heat through light irradiation. The heat energy is then used to trigger reversible structure changes of thermal-responsive hydrogels, subsequently cause drug release from the system (Pan et al., 2021/10). Some commonly used photothermal agents include both inorganic nanoparticles and organic compounds, such as rare metal nanostructures and black phosphorus (BP) nanoparticles, which have little phototoxicity. These photothermal agents have minimized damage to cells and good penetration for high efficiency, and thus can provide safe thermal effects for controlled drug release (Zhang et al., 2015; Zhu et al., 2015; Wang et al., 2016; Yang et al., 2018). Commonly used thermal-responsive hydrogels include poly(N-isopropylacrylamide) (PNIPAAm), thermosensitive PEG analogs and thermosensitive elastin peptides (ELPs) (Ward and Georgiou, 2011). Because high temperature can lead to cell necrosis, the parameters of applied light irradiation should be carefully selected to avoid thermal damage to surrounding areas of targeted cells or tissues (Yang et al.eng, 2017). For this reason, NIR light is widely used to initiate photothermal effects because of its low energy and deep penetration properties. The drug release parameters can be accurately controlled by light duration and intensity, concentration of photothermal agents, and hydrogel composition (Merino et al., 2015).

BP nanoparticles are one of the most popular photothermal agents. Qiu et al. developed a low–melting-point agarose drug delivery nanostructure containing BP. After injected into cancer tissue, the hydrogel experiences a phase transition to gel states at body temperature. Under NIR irradiation, the hydrogel entered in a melting state which caused drug release from its matrix. Additionally, the release rate is able to be accurately controlled (Qiu et al., 2018). The system demonstrated a high therapeutic efficacy for cancers, and it is harmless and degradable *in vivo*. In another study, an injectable, NIR-II light-modulated and thermosensitive hydrogel is synthesized through supramolecular self-assembly of a conjugated polymer and alpha-cyclodextrin. This hydrogel intrinsically features NIR responsive characteristics and thermo-responsive properties (Figure 1H) (Ruan et al., 2019).

Rare metals are also widely used as photothermal agents. In one study, platinum nanoparticles were integrated into a NIR light-responsive hydrogel consisting of αCD and PEG-modified dendrimer. Under NIR irradiation, this hydrogel underwent a disruption to release the encapsulated drugs in a controlled manner *via* the irradiation time (Wang et al., 2016). Platinum nanoparticle has a very good photothermal conversion efficiency and biocompatibility; thus, it is frequently used with hydrogels for drug delivery. In another similar study, aldehyde-modified dextran hydrogel containing dendrimer-encapsulated platinum nanoparticles were developed *via* imine bond formation. The hydrogel exhibited excellent biocompatibility, photothermal effect and biodegradable properties. It can stay in tumors for days to allow repeated drug release, resulting in tumor regression (Li et al., 2018b).

For a study using organic compounds as the photothermal agent, one type of PNIPAM hydrogel has been synthesized with protoporphyrin IX or pheophorbide as photothermal agents, which are covalently conjugated to the polymer chains. The hydrogels showed great biocompatibility with more than 90% cell viability even at very high photosensitizer concentration suggesting the hydrogels can be applied for photothermal therapy (Belali et al.eng, 2018). In another study, the authors designed a novel type of dynamic-covalent hydrogel (GelPV-DOX-DBNP) for combined chemical and photothermal therapy of cancers. Anticancer drug DOX and photosensitizer perylene diimide zwitterionic polymer (PDS) as well as reductant ascorbic acid (Vc) were encapsulated. Under 600 nm light irradiation, PDS and VC can turn oxygen to hydrogen and cause degradation of hydrogel, subsequently lead to DOX and DBNP release from hydrogel. DBNPs are able to generate heat under NIR irradiation, making the system a useful drug delivery platform (Sun et al.eng, 2020). Cao et al. developed a chemo-photodynamic therapy system using ROS sensitive structure and successfully used for cancer treatment (Figure 1I) (Cao et al., 2018).

## 6 Light-induced multi-stimuli responsive drug delivery systems

Multi-stimuli responsive hydrogels have been used in a variety of physiological or pathological conditions. Besides responding to light, hydrogels can also be designed to respond to pH, magnetic field and reductant etc (Sharifzadeh and Hosseinkhani, 2017; Pham et al., 2020). In one study, hydrogels that can respond to both pH and NIR is designed to release adamantane-modified doxorubicin (DOX) prodrug using N-isopropylacrylamide (NIPAm) and βCD-based hydrogel. The pH-responsive release of DOX from the nanocomposite hydrogel was observed owing to the cleavage of acid-labile hydrazone bond between DOX and the adamantyl group in acidic environment. NIR irradiation led to accelerated release of DOX from hydrogels because of photothermal effects. The hydrogel can respond to both pH and NIR light and speed up drug release rate in a controlled manner (Xu et al.eng, 2017). In another study combining light and magnetic field, the authors designed a temperature-responsive PNIPAm hydrogel microfibers with controlled shapes and sizes. Then they fabricated light-responsive materials by incorporating photothermal magnetic nanoparticles within the PNIPAm microfibers. The magnetic nanoparticles were incorporated into the PNIPAm microfibers and created heat when subjected to visible light exposure. Volume changes of the PNIPAm hydrogel can be induced by both light irradiation and temperature, suggesting its potential use for drug delivery (Lim et al., 2015). Shao et al. developed a PEG based nanocarrier with light and redox dual responsive properties for cancer therapy. The system possesses good drug loading capacity and stability, and showed preferred tumor accumulation and the prolonged tumor residency by *in vivo* and *ex vivo* experiments (Shao et al., 2014). In another study, photo, pH and redox multi-responsive nanogels were developed for drug delivery and fluorescence cell imaging (Chen et al., 2017) (Figures 1J, K). Multi-stimuli responsive hydrogels are multi-functional, and suitable for multi-step drug delivery systems especially for complicated *in vivo* studies.

## 7 Discussion

Light is a powerful trigger for controlled drug delivery systems. Intensity, spatial and temporal control of light allows excellent manipulation of therapeutic agents in comparison with other physical, chemical, and biological stimuli (Chen et al., 2020). So far, much progress has been made in developing of innovative light responsive hydrogel drug delivery systems with both breadth and depth. But translating these studies into clinical applications still poses a significant challenge. Major issues need to be addressed includes: 1) most tumors are deep within the body, and it is difficult to deliver drugs to these locations. 2) the *in vivo* biological conditions of human are complex, therapeutic effects of drugs are low comparing *in vitro* or animal studies. 3) biomaterials of the drug delivery system, including hydrogel, photosensitizer, drug carrier and other components, may accumulate within human body after long-term administration. 4) most light induced drug delivery systems are irreversible, which made drug release inconsistent during the process. 5) non-specific photoreactions in normal tissues need to be considered. 6) photosensitizers are still active in dark conditions, and thus stability of loaded drugs cannot be maintained especially for long-term use.

Considering above issues, potential future developments may include: 1) designing photosensitizers responsive to low energy light source with fast response, high efficacy and deep tissue penetration (Li et al., 2019). Low energy lights, such as NIR, also cause less non-specific phototoxicity in normal tissues. 2) characterizing *in vivo* biological conditions, such as pH, temperature, redox and enzymatic reactions. These stimuli should be used together with light for drug release. 3) developing highly biocompatible materials for drug delivery systems. Natural biomaterials that mimic human tissues may be promising. 4) combing photoisomerization with other reactions to design reversible systems. Light-responsive proteins may have wider applications for this purpose (Tao et al., 2020). 5) besides using low energy light, monitoring real time drug distribution within tissue is another way minimize non-specific photoreactions. 6) developing stable photosensitizers for long-term stable drug release. Additionally, combining two or more independent photo-induced reactions into one drug delivery system is appealing for clinical use (LeValley et al.eng, 2020).
